# Isolation, characterization and expression in *Escherichia* coli of a cDNA coding for a novel major wheat food allergen

**DOI:** 10.1186/2045-7022-1-S1-P97

**Published:** 2011-08-12

**Authors:** Alexandra Schmidhuber, Sandra Pahr, Claudia Constantin, Nikos Papadopoulos, Chritof Ebner, Adriano Mari, Susanne Vrtala, Rudolf Valenta

**Affiliations:** 1 Christian Doppler Laboratory for the development of allergen chips, Department of Pathophysiology and Allergy Research, Medical University of Vienna, Vienna, Austria; 2 Department of Patholphysiology and Allergy Research, Medical University of Vienna, Vienna, Austria; 3 Allergy and Immunology Research Centre, University of Athens, Athens, Greece; 4 Ambulatory for Allergy and Clinical Immunology, Vienna, Austria; 5 Centre for Clinical and Experimental Allergology, IDI-IRCCS, Rome, Italy; 6 Christian Doppler Laboratory for the development of allergen chips, Department of Pathophysiology and Allergy Research, Medical University of Vienna, Vienna, Austria; 7 Christian Doppler Laboratory for Allergy Research, Department of Pathophysiology and Allergy Research, Medical University of Vienna, Vienna, Austria

## Introduction

Wheat (*Triticum aestivum*) is a main component of the daily diet but can cause three distinct forms of wheat allergy: Baker's asthma, wheat food allergy and wheat pollen allergy. The panel of wheat allergens is still incomplete. The aim of the study was to identify and characterize wheat allergens for the development of improved diagnostic tests and allergen-specific forms of treatment.

## Methods

A cDNA library was screened with serum from wheat food allergic patients. The cDNAs coding for allergens were subjected to sequence comparison, cloned into *E.coli* expression vectors and recombinant allergens were purified. The IgE reactivity of the recombinant allergens was tested by non-denaturing RAST-based dot blot analysis with sera from clinically well defined patients suffering from wheat food allergy or Baker's Asthma.

## Results

We isolated a cDNA coding for the C-terminal part of a low molecular weight glutenin which has not yet been described as an allergen. The C-terminal part as well as the full length protein were expressed as soluble proteins in *E. coli* and purified. More than 80% of wheat food allergic children (n=26) showed IgE reactivity with the complete recombinant glutenin whereas only 5% of Baker's Asthma patients (n=60) showed specific IgE reactivity.

## Conclusion

We identified a low molecular weight as a novel major wheat food allergen which can be used for the development of component-resolved diagnostic tests for wheat food allergy and eventually for specific immunotherapy.

